# Mental Epidemics

**Published:** 1881-07

**Authors:** 


### MENTAL EPIDEMICS.

There are times when a whole community is ravaged by a particular disease or pestilence or plague; at one time they sweep along like a whirlwind and all is over in a night, at others they move slowly through days and weeks, and months, as resistless as an avalanche. The cholera, the plague, and the black death, have all had their day. But it is not as generally recognized that at different periods in the world's history, mental epidemics have had their sway, as resistless and as wide, and full as hurtful to the moral, as plagues have been to the physical world, as blighting in their influence on the mind as the black death or the Asiatic cholera were to the body. Mohamedanism, the New Lightism of Kentucky at the opening of the present century, when at Cane Ridge in Bourbon county near Paris, men and women as the first steps toward "conversion," at one time would be taken with "a falling," and would roll and tumble about on the ground with uncontrollable violence; another man in a different crowd would be "taken with a barking," and forthwith a dozen others would imitate for hours the barking of dogs. In later times, so recently as a third of a century ago, the whole Western country was overrun with the "*anxious seat*" furor. At the close of a sermon or religious address, all persons, who were not members of the church, and felt any anxiety whatever to become religious, were invited to take a specified seat, usually the one nearest the pulpit, in order "to be prayed for;" and "while a hymn was singing," to employ the current phrase, some one, who perhaps was least expected to have any solicitude in that direction, would rise in the congregation, and with every eye upon him, would wend his way among the dense and expectant crowd; and reaching the spot, would fall helplessly in his seat, and with a convulsive sob, yield himself to uncontrollable grief; in another instant, dozens, and scores, and hundreds, as I myself have seen, would make a rush to the *anxious seat*, creating such an influence, such a presence, as it were in the assembly, that saint and sinner were dissolved in tears. And any Sunday in New Orleans, you may now see, in the African church, a species of mental epidemic wholly impossible of counterfeiture. One day I saw a likely young man, colored, bend himself backward in the shape of a half hoop, and remain as stiff in limb and turgid in muscle for many minutes, as if he were in an uncontrollable fit; while young women would jump up and down, crying "amen," "glory," "hallelujah," or some other scripture word, for a length of time wholly impossible for any one in his right mind; and this would at times extend from one to another, until there were dancers all over the house.

*Mormonism* may be well regarded as another of those mental epidemics, but of far more pernicious tendencies, judging by the first fruits and thus far. As of this same class, we may mention millerism, mesmerism, biology, spirit rapping, table turning and mediation, more hurtful in their tendencies than even mormonism, in their present state of explication: there is so much that is conjectural, contradictory and intangible, as to make wise men mad, let alone the greater multitude of the weaker sort.

It is usual, when epidemical diseases make their appearance, to appoint the most competent and capable among physicians to observe, analyze and report, and then to adopt such a course of experiment as may seem best adapted to elicit truth, and to save human life. And it seems to me, that a like course would have resulted in great practical good in the recent mental or moral epidemics. But this has not been done. The clergy as a body have stood aloof, or with elevated hands and uplifted eyes, have cried, "*procul, procul, este profani.*" The funny minded have poked their fun; the loafing multitude, too lazy to examine, have exclaimed, "*it's all a humbug;*" while young editors of newspapers, or fledgeling editors of young newspapers, regarding it as a godsend for the exhibition of their wit and wisdom, have used by turns anathema and ridicule; while all this time the weaker multitude are wondering, and then won, and alas! in many instances, to their own undoing, in soul, body and estate.

"Humbug," has been the general cry as to spiritualism. But humbug is the argument alike of the ninny and the knave, of the loafer and the numscull; it is the argument of those who are too lazy to investigate, or too weak-minded to comprehend, reason and decide. The exclamation "humbug," is no argument, proves nothing, and yet may accomplish a great deal—as potent sometimes as "persecution." While this cry has been sounded and echoed and re-echoed from plain to mountain top over the breadth of this wide land, Joe Smith, and Miller and Miss Fox have rallied their hosts and counted their followers by scores of thousands, and whether it shall reach to hundreds of thousands no man can tell.

In the earliest ages of christianity, the great argument against it, among the aristocratic learned in Greece and Rome was, contemptuous derision; and even earlier, it was considered irrefutable to inquire, "*Can any good come out of Nazareth?*" Can a few ignorant fishermen propose any thing deserving our consideration; and when now and then the truths of religion swept with resistless power over the intellect and heart of some one of their own literati, more candid than his fellows, the force of the fact was attempted to be broken by the summary exclamation, "*He's crazy!*" "*Much learning hath made thee mad!*" We have an exact counterpart in the history of spiritualism in our own day and generation, and with a like result too—the conversion of multitudes.* And yet, the men who write editorials for religious newspapers follow in the wake of the contemptuous abusers of primitive christianity, and write about spiritualism with such an evident impatience of temper, with such palpable want of forbearance, as may well evoke the inquiry: Is this the spirit of truth? Is this the *animus* of a true learner?

The first error which the educated have committed in reference to spiritualism was, in denying too much; they adopted the line of policy of a weak pettifogger, conscious not only of his own weakness, but of his cause also—admitted nothing and denied every thing. When a sentiment was announced which was altogether adverse to their previous views, to opinions cherished from childhood, they would break out with an exclamation, "The idea!" *How preposterous! It's absurd. I never heard of such a thing.* Very likely it was an idea, and might be preposterous and absurd, and it is reasonable to suppose they might not have heard of such a thing, but there was no argument in any of these assertions. For a long time it was denied that there were knockings except by collusion, and that the movement of tables could not occur except by deception, and yet I believe it is taken for granted now by all who have the patience and ability to investigate. The same contemptuousness was exhibited in relation to Morse's telegraph, towards Fulton's steamboat, and to all great new truths. That being the case, men, capable of investigating abstruse things, should have known that a sneer is, if possible more powerless against error than against truth.

I own myself to a feeling of pitying contemptuousness towards persons the moment I hear of their leaning towards spiritualism, just as I do towards a man when I see him voluntarily place his "head" under a phrenologist's fingers, and I often feel sensible that I am thus doing them an injustice and seeing the inconsistency, I never deride, never contemn in audible words; for this is not the spirit which animates an

* Judge Edmonds, before he avowed spiritualism, was considered an able lawyer; and not many men on the bench have had so few of their decisions reversed in the superior courts; and yet the moment he became a spiritualist—"*his head is turned!*"

honorable and candid inquirer after truth. I have lived long enough to have embraced things which I once laughed at, and feel that at some future day I may admire what I now consider an absurdity or an impossibility. Hence, I like modest believers. Violent advocates are much like raw soldiers in a first battle, very apt to turn about, and march rather more vigorously from, than forwards.

There is a practical lesson which I wish to inculcate by the above remarks, for two very different classes of people.

1st. To those who are not accustomed to investigate abstruse points, I commend the injunction of scripture in reference to another subject, "*Touch not, taste not, handle not.*" Have, just now, nothing to do with spiritualism, mormonism, Millerism. "Let alone" is the best policy for ordinary minds. Do not go to their meetings, always avoid argument with their adherents, never allow any of their books, or papers or publications to come inside your doors, anymore than you would the writings of Tom Paine, Lord Byron, or Percy Bishy Shelly; no more than you would allow a man to come daily to your house and sell your children sweetened whiskey punch or brandy toddy. The more these things abound, the more should you read the Bible, the Prayer-book, the Confession of Faith, and the sturdy old sermonizers of a hundred years ago. In these days of making many books, of gilded infidelity and painted corruption and rottenness in things mental and moral, the great line of safety is, *keep out of harm's way*, go not into the way of temptation, tamper not with a possible lurking viper. Common people—wait for the light! pitfalls are all around you, and groping for yourselves, fathomless abysses will receive you.

But really I have spent an aimless arrow. I have appealed to the *common* people; but as not a man, woman, or child in this free country, believes that he she or it is a common person, I must aim again, and hit two birds with one stone, by suggesting the second practical lesson to the uncommon people of this broad land, to the intelligent, to the thoughtful, to the candid, to those who are capable of abstruse investigations, who can examine Spiritualism or any other ism, with a mind open to conviction, not hampered with the firm belief that it is all an imposition, and with a determination never to be convinced; to the noble few who are not afraid to admit a truth, however it may subverse present opinions or previous avowals; to that class of brave minds who are willing to follow wherever truth may lead, be it to the cannon's mouth, be it to the stake! to such I desire to offer a suggestion, that as it is a duty of the bodily strong to use their strength when the house is on fire, or the ship is sinking, to save the weaker ones, the children, and women, and sick; and if they do not do it, the contempt and curses of a whole community are hurled heapingly upon them—as for example the Arctic catastrophe!—So in the mental and moral world, the strong-minded, the educated, the men of thought and leisure, are as much bound to do a duty, and as much deserve execration, if it is not done. That duty is to put forth their strength in saving their weaker brethren from delusions worse than death: for what American mother would not rather see her daughter die, than be the fortieth wife of a Mormon, or to be the crazy dupe of Millerism, or the demented raver after the crude vagaries of intangible Spiritualism? In this age of activity, when nothing waits, are competent men to fold their arms, when mischievous errors are promulgated, and say, "Oh! it is all a delusion, and will die the sooner from its being let alone?" But the fact is, if such even let it alone, the multitude will not, hence its spread; but the greater absurdity is to make a starting point by assuming as a truth what all Spiritualists deny, and unnumbered thousands of the common people deny too, to wit: that Spiritualism *is* an absurdity. That is the point to be proven, and men of mind ought to have accomplished it long ago, if possible, and thus have arrested in the bud an asserted evil. The plainer the absurdity, the easier of proof, it seems to me. And to my mind, the only way of proving the absurdity of any sentiment or assertion, is to take point by point, specification by specification; meet it by matured, serious, sterling argument, and let it be so conclusive, that the commonest mind may see it almost by intuition. It is not an easy matter to pick up any "*Religious Newspaper*," so called, of any denomination, among protestants, and find anything on the subject of Mormonism, Millerism, Spiritualism, *et id omne*, which does not bear upon its face ample evidence of impatient invective. Common minds feel this, see its emptiness of argument, and finding no light from sources which ought to afford it; they do the next best thing they can, and grope and flounder along in darkness visible, through seas of argument, as clear as mud, and not less defiling.

Gentlemen in black, I think verily, that you are not using your Master's capital wisely! You make investments which enable you to hew and hack each other right well, Protestant against Puritan, and both against Pope, and *vice versa;* but as to affording the common people light to see the absurdities of the isms already named; I think you are where boys in the grammar-school are said to be, when books were neglected for play,—*in the vocative*,—"*wanting*."

But to all I say, if you feel called upon to investigate any of the "*new lights*" of the times, and have a desire that you should not soon find yourselves "in endless mazes lost," there is but one insurance office against so sad a calamity in this wide, wide world, and that is the Bible; make it your guide, let it be your Polar star, plant your foot immovably on its clearly asserted principles; principles which King James' translation fully warrants; principles fairly, clearly deducible from the English text, as it is, without the mischievous "*better renderings*," which sophomore clergymen are so often and so mischievously talking about. I say, stand upon the platform of such principles, backed, as they must be, by the great and good in all past ages, and no fear, I tell ye, that the immortal bark shall founder on shore, or breaker, or sunken rock; but passing them all triumphantly, it will find secure anchorage in the haven of everlasting rest.

